# 肺癌术后特发性肺纤维化急性加重抢救成功病例报道

**DOI:** 10.3779/j.issn.1009-3419.2022.102.13

**Published:** 2022-05-20

**Authors:** 川 黄, 青峻 吴, 超 马, 鹏 焦, 耀光 孙, 宏峰 佟

**Affiliations:** 100730 北京，北京医院胸外科，国家老年医学中心，中国医学科学院老年医学研究院 Department of Thoracic Surgery, Beijing Hospital, National Center of Gerontology, Institute of Geriatric Medicine, Chinese Academy of Medical Sciences, Beijing 100730, China

**Keywords:** 肺肿瘤, 肺切除术, 特发性肺纤维化, 急性加重, Lung neoplasms, Idiopathic pulmonary fibrosis, Acute exacerbation, Pneumonectomy

## Abstract

特发性肺纤维化（idiopathic pulmonary fibrosis, IPF）是一种病因不明、慢性进行性、纤维化性间质性肺病，与肺癌关系密切，肺切除术后易出现IPF急性加重（acute exacerbation of IPF, AE-IPF），病情进展迅速，缺乏有效治疗，预后极差。本文报道1例肺癌术后AE-IPF典型病例，总结其诊治要点，以提高临床医师对肺癌手术后AE-IPF的重视。

## Abstract (en)

Idiopathic Pulmonary fibrosis (IPF) is a chronic, progressive, fibrotic interstitial lung disease with unknown cause, which is closely related to lung cancer. A serious complication called Acute exacerbation of IPF (AE-IPF) is prone to occur after lung resection. It progresses rapidly without effective treatment and has a poor prognosis. A typical case of AE-IPF after lung cancer surgery was reported, and its clinical characteristics, imaging features, diagnosis and treatment were summarized.

## 1 一般资料

### 1.1 病例资料

患者男性，60岁，体检发现右肺上叶结节，平素间断干咳，无咳痰、咯血、胸痛、发热等不适，双肺野外周可闻及少量干啰音，余无异常。

既往史：30年前当防化兵时曾长期接触有毒化学物质，发现特发性肺纤维化（idiopathic pulmonary fibrosis, IPF）病史20余年，呼吸科规律随诊，每年复查胸部计算机断层扫描（computed tomography, CT）显示IPF病情缓慢进展，未用药。2型糖尿病史18年，平素规律使用降糖药物+胰岛素，血糖控制良好。冠状动脉粥样硬化型心脏病史5年，冠脉造影显示左前降支、右冠状动脉轻度狭窄，口服阿司匹林（100 mg/d）、降脂药物。吸烟史50年，3包/d，戒烟1周，否认饮酒史。

完善相关检查，肺功能：第1秒用力呼气量（forced expiratory volume in the first second, FEV_1_）2.05 L，FEV_1_占预计值百分比（FEV_1_%）69%，用气肺活量（forced vital capacity, FVC）2.54 L，FVC占预计值百分比（FVC%）66%，FEV_1_/FVC 80%，肺活量百分比（vital capacity, VC）67%，一氧化碳弥散量（diffusion capacity for carbon monoxide of lung, DLCO%）67%。动脉血气：动脉血氧分压（partial pressure of oxygen, PaO_2_）93 mmHg，动脉血二氧化碳分压（partial pressure of carbon dioxide in artery, PaCO_2_）38 mmHg，氧饱和度98%。腹部超声、头颅核磁、骨扫描等肿瘤分期检查未见远处转移（图 1）。

**图 1:**
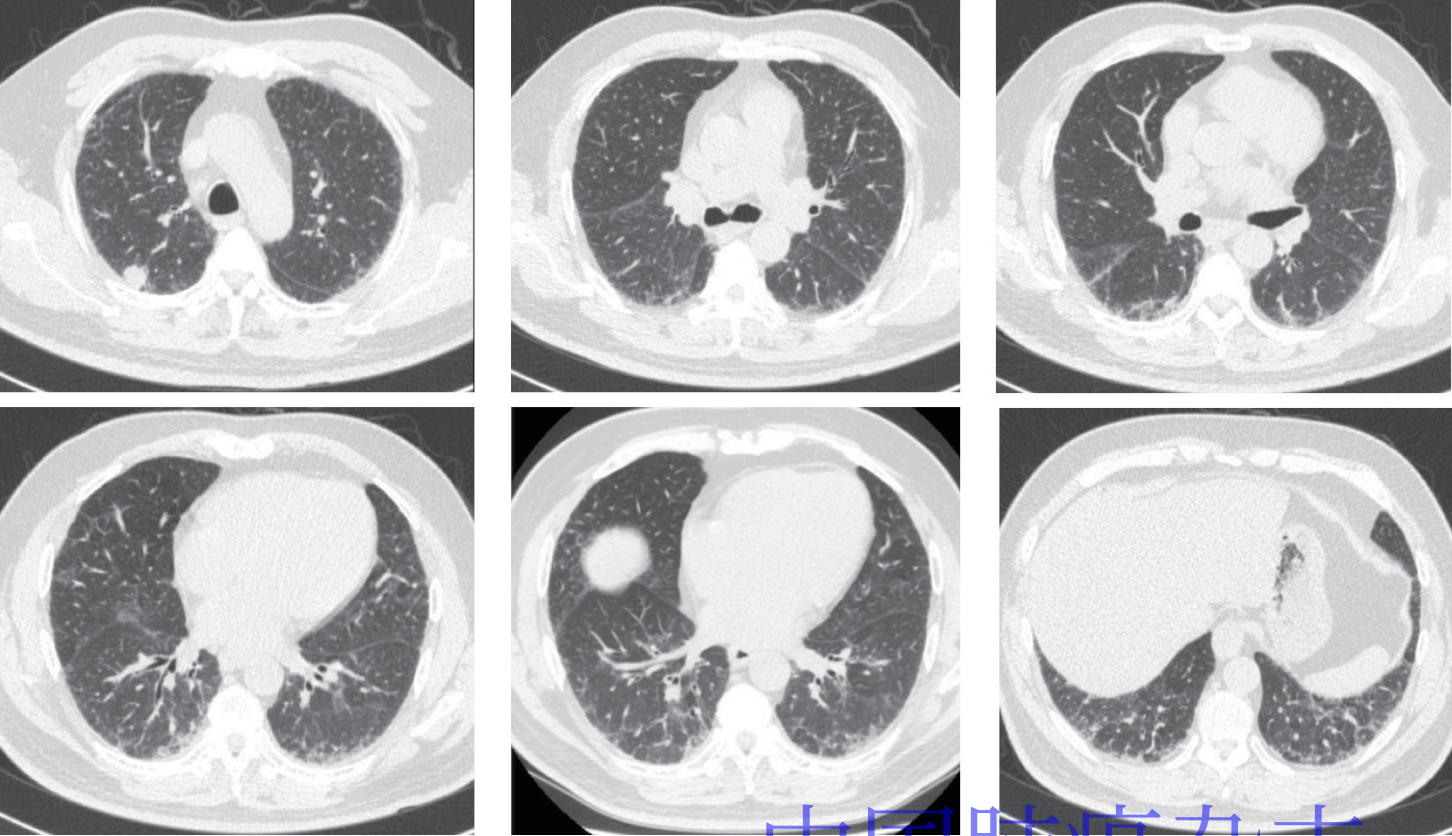
胸部CT（术前）：右肺上叶胸膜下类圆形结节影，直径1.6 cm；双肺多发小叶间隔增厚、磨玻璃影、网格影及索条影，以双下肺为著，符合双肺间质纤维化。 Chest CT (preoperative) : subpleural nodules in the upper lobe of the right lung, 1.6 cm in diameter; There were multiple interlobular septal thickening, ground glass shadow, mesh shadow and cable shadow in both lungs, especially in both lower lungs. CT: computed tomography.

手术方式：2015年10月14日在全身麻醉下行单操作孔胸腔镜右肺上叶切除、系统性淋巴结清扫术，手术顺利，病理为小细胞肺癌，侵犯脏层胸膜，第10组淋巴结可见转移癌，病理分期：pT2aN1M0，Ⅱb期。

### 1.2 术后转归

术后第1天患者病情平稳，少量咳痰，无明显胸闷、憋气，顺利恢复饮食、下床活动，体温正常，双肺野外周可闻及少量散在湿啰音，余肺野呼吸音清，脉搏氧饱和度（pulse oxygen saturation, SpO_2_）为95%-100%。胸片（图 2A）显示双肺透光度减低，肺纹理增粗、紊乱。

**图 2:**
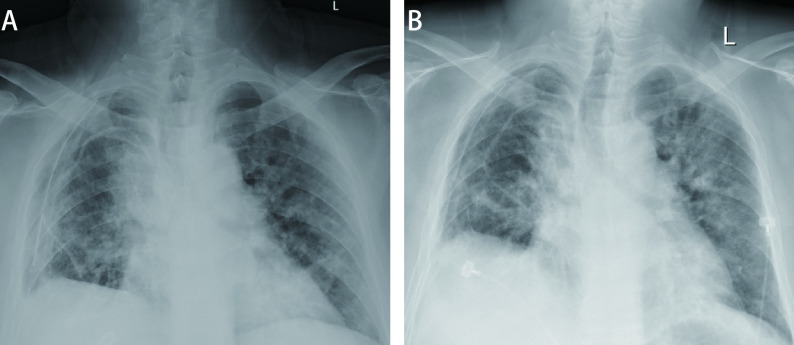
胸片。A：术后第1天上午，床旁坐位胸片，双肺透光度减低，双肺纹理增粗；B：术后第3天上午，床旁坐位胸片，双肺透光度减低，双肺纹理增粗。 Chest radiograph. A: On the morning of the 1^st^ postoperative day, chest radiograph at bedside showed reduced translucency and thickening of both lungs; B: On the morning of the 3^rd^ postoperative day, chest radiograph at the bedside showed reduced translucency and thickened texture of both lungs.

术后第2天患者胸闷、气短、心悸症状逐渐加重，咳少量黄白色粘痰，SpO_2_为90%-96%，双肺湿啰音较前明显、范围增大。予以床旁协助患者拍背咳痰，雾化吸入3次/d，抗生素升级至哌拉西林舒巴坦，强心、利尿，皮下注射低分子肝素抗凝，术后输液限速≤150 mL/h，控制输液量，术后第1天入量3, 395 mL、尿量1, 600 mL、胸腔引流量400 mL，第2天入量2, 865 mL、尿量1, 850 mL、胸腔引流量400 mL，第3天入量1, 950 mL、尿量1, 000 mL、胸腔引流量400 mL。

术后第2天夜间至第3天上午，患者咳嗽增多，咳白黏痰，呼吸困难进展迅速，双肺野可闻及广泛吸气末爆裂音，体温最高39.5 oC，无寒战，心率95次/min-110次/min，鼻导管吸氧难以维持，储氧面罩吸氧状态下SpO_2_仅达88%-94%。动脉血气：PaO_2_ 54 mmHg，PaCO_2_ 34 mmHg。血常规：白细胞13.62×10^9^/L，中性粒细胞百分比78.4%，嗜酸性粒细胞百分比2%。B型钠尿肽（brain natriuretic peptide, BNP）421 pg/mL（正常 < 125 pg/mL）。复查胸片（图 2B）显示双肺透光度较前减低，双肺纹理较前增粗。

经过上述氧疗、祛痰、强心、利尿等治疗，患者呼吸困难不仅未能缓解、反而迅速加重。为协助诊断，急诊行胸部CT（图 3）显示双肺多发小叶间隔增厚、磨玻璃影、网格影及索条影，为新出现，以双下肺为著。

**图 3:**
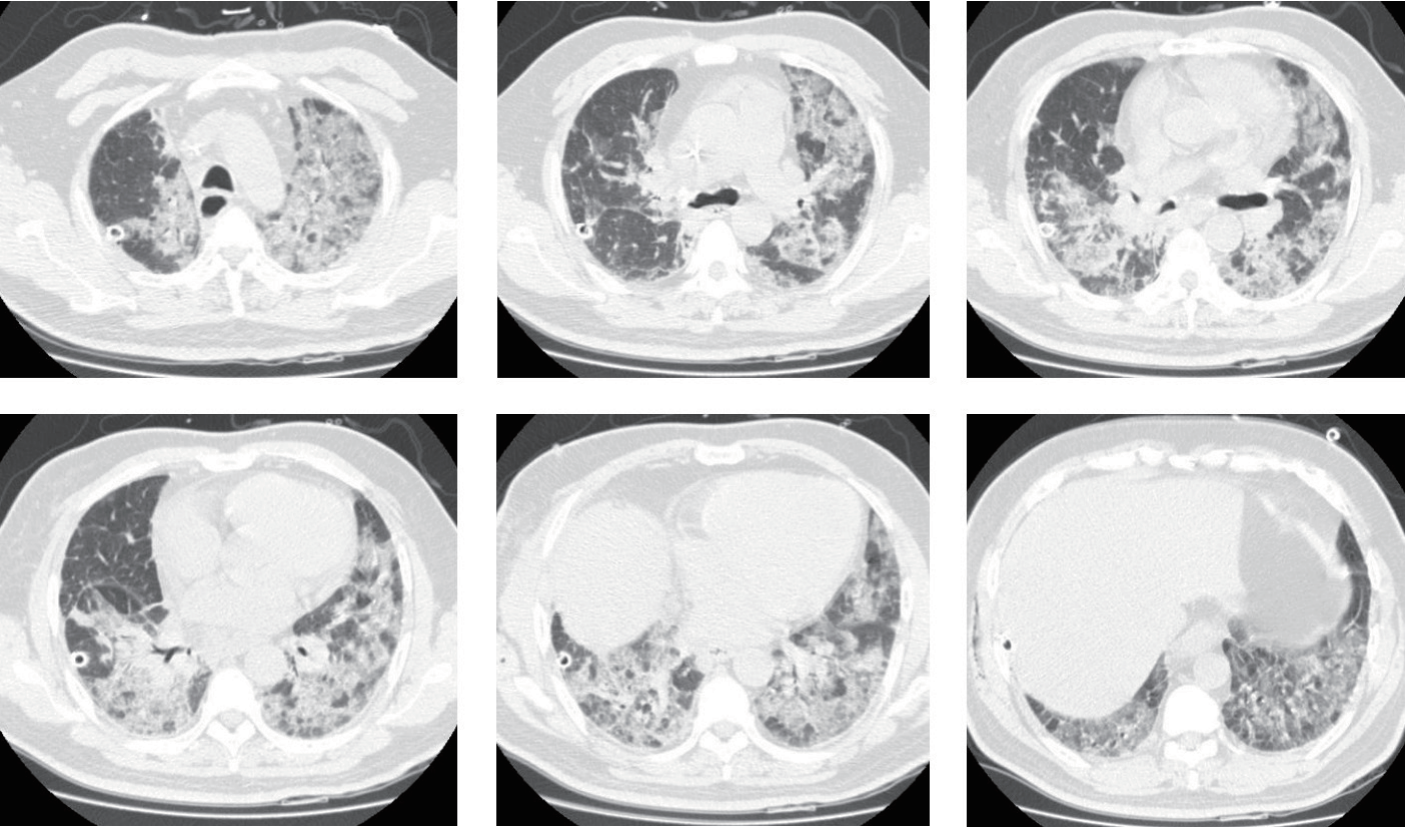
胸部CT（术后第3天）：双肺多发小叶间隔增厚、磨玻璃影、网格影及索条影，为新出现，以双下肺为著。 Chest CT (postoperative day 3) : Multiple thickening of interlobular septa in both lungs, ground glass shadows, grid shadows and cable shadows, which appeared newly after surgery, especially in both lower lungs.

### 1.3 诊断与鉴别诊断

#### 1.3.1 诊断

特发性肺纤维化急性加重；Ⅰ型呼吸衰竭；右肺上叶小细胞癌；右肺上叶切除术后；糖尿病；冠心病。

诊断依据：患者既往有明确IPF病史，术前IPF病情稳定，肺切除术后2天-3天内出现快速进展的呼吸困难，双肺可闻及吸气末爆裂音，胸部CT显示在原有IPF影像改变基础上出现新的、双肺分布的小叶间隔增厚、磨玻璃影、网格影及索条影，以上符合IPF急性加重（acute exacerbation of IPF, AE-IPF）的诊断标准。

#### 1.3.2 鉴别诊断

##### 1.3.2.1 肺部感染

肺切除术后可因咳嗽咳痰无力、气道分泌物潴留、肺阻塞不张等因素导致肺部感染，气道分泌物可查到细菌。本患者术后咳痰有力，无气道分泌物潴留，胸片和CT未见肺不张，治疗过程中反复留取气道分泌物涂片和细菌培养，均未见细菌，可以排除肺部感染。

##### 1.3.2.2 左心衰、心源性肺水肿

心功能差的患者行较大范围肺切除术后，可因输液速度过快、循环容量过负荷导致心功能不全，诱发心源性肺水肿，可表现为呼吸困难、Ⅰ型呼吸衰竭。本患者虽合并有冠心病，但术前心功能尚好，围术期输液速度管理严格，无循环容量过负荷，病情加重时复查心功能指标基本正常，可以排除急性左心衰导致的肺水肿。

##### 1.3.2.3 急性肺栓塞

外科大手术后可因血管内皮损伤、凝血功能亢进、使用止血药物、长期卧床等因素增加深静脉血栓、急性肺栓塞发生率，可表现为急性呼吸困难、Ⅰ型呼吸衰竭。本患者围术期未使用止血药物，术后抗凝治疗及时，无长期卧床，下地活动良好，病情加重时复查血D-二聚体无明显升高，基本可以排除急性肺栓塞。

综合以上，经胸外科、呼吸与危重症医学科、影像科的多学科讨论，诊断为AE-IPF。

### 1.4 治疗情况及治疗转归

#### 1.4.1 治疗情况

为改善急性肺间质炎症，术后第3天上午开始糖皮质激素治疗，方案：甲泼尼龙琥珀酸钠静脉滴注（第1天40 mg/d，第2天80 mg/d，第3天160 mg/d，第4天-5天120 mg/d，第6天-第8天80 mg/d，第9天-第11天60 mg/d，第12天-第23天40 mg/d），此后改为醋酸泼尼松口服（40 mg/d，每2周减量5 mg），最终于术后3个月将口服激素减量至停药。

治疗期间使用无创呼吸机辅助通气4天，呼吸改善后改用储氧面罩吸氧，病情稳定后改用鼻导管吸氧。比阿培南、盐酸莫西沙星抗感染治疗1周，期间反复行痰细菌培养均为阴性，随后停用抗生素。

#### 1.4.2 治疗转归

患者呼吸困难逐渐缓解，双肺野爆裂音减轻，病情明显改善。糖皮质激素治疗后2周、4周、3个月分别复查胸部CT（图 4、图 5、图 6）显示双肺间质炎症逐渐吸收、减少。最终患者恢复良好，术后第40天顺利出院，于肿瘤科完成术后辅助化疗。

**图 4:**
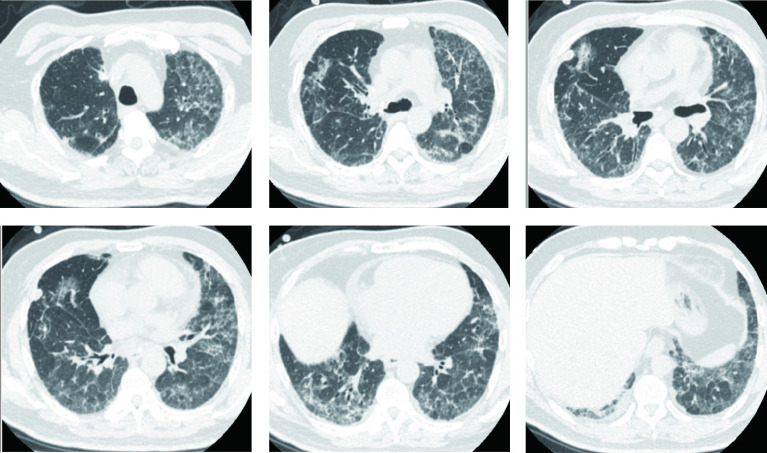
胸部CT（糖皮质激素治疗2周后）：双肺弥漫斑片状磨玻璃影较前减少。 Chest CT (after 2 weeks of glucocorticoid treatment): Diffuse patchy ground glass shadows in both lungs were significantly reduced compared to before treatment.

**图 5:**
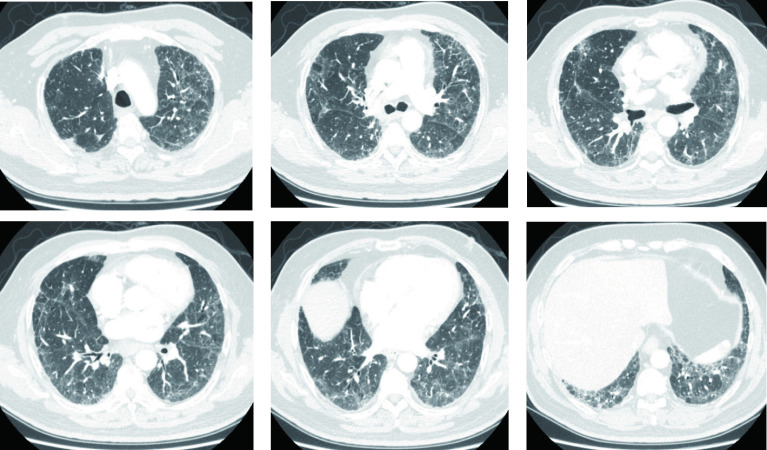
胸部CT（糖皮质激素治疗4周后）：双肺弥漫斑片状磨玻璃影较前明显减少。 Chest CT (after 4 weeks of glucocorticoid treatment): Diffuse patchy ground glass shadows in both lungs were significantly reduced compared to before treatment.

**图 6:**
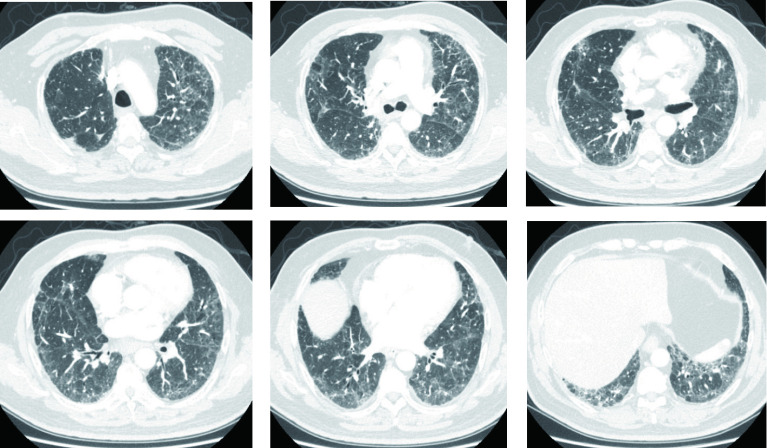
胸部CT（糖皮质激素治疗3个月后）：双肺弥漫斑片状磨玻璃影较前明显减少。 Chest CT (after 3 months of glucocorticoid treatment): Diffuse patchy ground glass shadows in both lungs were significantly reduced compared to before treatment.

## 2 讨论

IPF是一种病因不明、慢性进行性、纤维化性的间质性肺病，表现为进行性加重的呼吸困难，伴限制性通气功能障碍和气体交换障碍，导致低氧血症，预后较差^[1]^。IPF合并肺癌的发病率可达10%-17%，以男性、吸烟者居多，多发生于肺外周纤维化明显的区域^[2]^。本例患者有IPF病史20余年，胸部CT发现肺外周占位，应警惕IPF合并肺癌，微创手术切除肺部病灶则兼顾了诊断和治疗的作用。

通常IPF进展缓慢，但部分患者可在病情相对稳定的情况下出现病情急剧恶化，称之为AE-IPF，其最突出症状为快速进展的呼吸困难，肺部常可闻及吸气末爆裂音（velcro啰音），胸部CT表现为在原有IPF影像改变基础上出现新的、双肺分布的磨玻璃阴影或实变阴影，应注意排除肺部感染、左心衰、肺栓塞和其他原因引起的急性肺损伤。本例患者术前IPF病情稳定、缓慢进展，术后2天-3天内出现不明原因、快速进展的呼吸困难，伴顽固性低氧血症，相关检查可排除心衰、肺栓塞、肺部感染，结合胸部CT特点，符合AE-IPF诊断。

IPF合并肺癌患者行支气管镜检查和镜下治疗、肺活检、肺切除、胸部放疗等均可诱发AE-IPF^[3]^，发生率可达12%-27%，预后很差，死亡率高达30%-100%^[4]^。AE-IPF多发生于术后第2-第10天，不仅累及术侧肺，更有超过60%患者累及双侧肺，男性、既往曾发生过AE-IPF、肺切除范围大、胸部CT表现为典型肺纤维化、血清涎液化糖链抗原-6 > 1, 000 U/mL、FEV_1_%≤80%、肺弥散功能降低等均增加肺切除术后AE-IPF风险，肺叶、双肺叶、全肺切除术后AE-IPF发生率明显高于楔形切除^[5, 6]^。因此，IPF患者接受有创诊疗措施时应谨慎衡量AE-IPF风险，根据肺纤维化程度和心肺功能情况，合理选择术式和切除范围，尽量微创化、避免切除过多肺组织，降低手术风险。

值得注意的是，胸外科手术后AE-IPF的诊断难点在于不易早期发现，肺叶切除术后患者多有咳嗽、胸闷、气短症状，肺容积减少、气道分泌物阻塞、肺不张、手术部位疼痛、循环容量过负荷等因素均可导致明显的咳嗽、喘憋、呼吸困难、低氧血症等，临床表现与AE-IPF很类似，发病早期不易鉴别。而AE-IPF病情进展非常迅速，若不能及时诊断和干预，患者往往快速进展至呼吸衰竭、需机械通气，而此时难以再行胸部CT检查以协助诊断。因此，术前应详细询问IPF病史，结合胸部CT影像和肺功能指标，充分评估肺纤维化程度和进展速度，病情严重者提前用药干预，避免IPF病情评估不充分而极大地增加手术风险。手术后短期内出现的快速进展的呼吸困难，应及时针对AE-IPF做鉴别诊断，尽早行胸部CT明确诊断，避免延误诊断和治疗。

临床上常用激素冲击（甲泼尼龙500 mg/d-1, 000 mg/d）或高剂量激素治疗（泼尼松≥1 mg/kg/d）治疗AE-IPF，激素疗效不佳时，可加用免疫抑制剂（如环磷酰胺、他克莫司、环孢素A等）。本例患者确诊AE-IPF后经长疗程激素治疗，症状改善明显，影像复查显示肺间质急性炎症逐步消退，最终预后良好，属治疗成功案例。对于激素的使用，我们有以下体会：介入时机应早，一旦疑诊AE-IPF，当天就应使用激素；疗程应足，待患者症状充分改善且经影像学证实肺间质炎症彻底缓解，再考虑停药；减量应慢，病情改善后，静脉激素应逐渐向口服激素减量和维持，病情尚不稳定就过快减量或停药易导致肺间质炎症的再次加重，此时即使再增加激素用量，效果也往往不佳。因此，早期、足量、足疗程的糖皮质激素有助于改善AE-IPF病情，治疗期间应谨防病情反复。
